# Case Report: Human Bocavirus Associated Pneumonia as Cause of Acute Injury, Cologne, Germany

**DOI:** 10.1097/MD.0000000000001587

**Published:** 2015-10-23

**Authors:** Michael Krakau, Kathrin Gerbershagen, Ulrich Frost, Markus Hinzke, Michael Brockmann, Verena Schildgen, Axel Goßmann, Volker Limmroth, Arno Dormann, Oliver Schildgen

**Affiliations:** From the Kliniken der Stadt Köln gGmbH, Medizinische Klinik Holweide, Lehrkrankenhaus der Universität zu Köln, Köln, Germany (MK, AD); Kliniken der Stadt Köln gGmbH, Klinikum der Privaten Universität Witten/Herdecke mit Sitz in Köln, Neurologische Klinik, Köln, Germany (KG, UF, MH, VL, OS); Kliniken der Stadt Köln gGmbH, Klinikum der Privaten Universität Witten/Herdecke mit Sitz in Köln, Institut für Pathologie, Köln, Germany (MB, VS); and Kliniken der Stadt Köln gGmbH, Klinikum der Privaten Universität Witten/Herdecke mit Sitz in Köln, Radiologische Klinik, Köln, Germany (AG).

## Abstract

Although the human bocavirus (HBoV) is known since a decade, limited information about its pathogenesis is available due to the lack of an animal model. Thus, clinical cases and studies are the major source of novel information about the course of infection and the related pathophysiology.

In this context, a clinical case of an adult patient suffering from severe HBoV-pneumonia is described that was associated with loss of consciousness followed by acute rib fracture and subsequent neurological disorder.

Following initial global respiratory dysfunction the clinical respiratory symptoms recovered but the neurological symptoms maintained after weaning and intensive care in the stroke unit. During the initial phase, an acute active HBoV infection was confirmed by positive polymerase chain reactions from bronchoalveolar lavage fluid and serum.

The case further demonstrates that HBoV can cause severe pneumonia, induce secondary disease also in adults, and may be associated with neurological symptoms as previously assumed.

## INTRODUCTION

The human bocavirus (HBoV) was described in 2005 as a respiratory pathogen.^1^ Since then, several clinical studies and reports have shown that the virus is indeed associated with respiratory infections of the upper and lower airways, may persist and be associated with long-term diseases like lung fibrosis and cancer.^1–11^

The virus appears to bear a narrow host and tissue range, thus hitherto, no animal model has been identified and only a spare number of cell types have been found to be permissive for the virus. Consequently, all available information about the pathogenesis of the HBoV infection is based on clinical observations from single case reports or cohort studies. In this context, the presented case contributes with a piece to the general puzzle of the HBoV infection.

## PRESENTING CONCERNS

The patient was a 74-year-old Caucasian male. He was admitted to the hospital by emergency service; 3 hr before admission, the patient had called his wife to report respiratory symptoms and a slump, but when she arrived at home the patient was unconscious and not responsive. The first aid team reported that the patient was nonresponsive and showed a cranial hematoma.

In the emergency room, the patient was responsive, an occipital hematoma was diagnosed, and the patient was mechanically ventilated because of the global respiratory insufficiency. On admission to the hospital and during the course the patient was sub-febrile. The electrocardiogram revealed a sinus-bradycardia, accompanied by regular pupils and light reaction and a pleural effusion. No further acute findings were observed.

## CLINICAL FINDINGS

During bronchoalveolar lavage (BAL) sapling the bronchial mucosa showed nonpurulent signs of inflammation with low amounts of secretions.

Detailed diagnostics revealed that the patient suffered from a spastic tetraparesis of unknown etiology, a severe pneumonia caused by HBoV, a cardiac decompensation, and a fresh rip fracture likely derived from the initial slump.

On laboratory investigations no further facultative or obligate respiratory pathogens (including virus, fungi, and bacteria) were detected by Respifinder Smart 22 and Meningofinder Custom Assays (Pathofinder, Maastricht, The Netherlands), *Pneumocystis jirovecii* polymerase chain reaction (PCR), nor by conventional microbiological screening methods. In detail, the patient's BAL was negative for influenza viruses, parainfluenza viruses 1 to 4, respiratory syncytial virus, human metapneumovirus, coronaviruses NL63, OC43, 229E, and HKU-1, adenoviruses, *Mycoplasma pneumoniae*, *Mycobacteria*, mumps, measles, humans herpesviruses 1 to 8, parechoviruses, rhinoviruses, enteroviruses, *Legionella pneumoniae*, *Chlamydia pneumoniae*, *P jirovecii*, *Aspergillus*, and *Bordetella pertussis* by molecular assays and also negative by culturing.

The sole pathogen detected in the BAL was HBoV and the active and acute infection was confirmed by a positive PCR against HBoV from a serum sample that was taken 2 days after the BAL was sampled. Unfortunately, it was not possible to isolate the virus from clinical specimen as cell culture maintenance was inhibited.

Besides the pneumonia, the patient developed massive pleural effusions and suffered from neurological disorders such as spastic tetraparesis of unclear origin.

## RADIOLOGICAL FINDINGS

The cranial computed tomography (CT) revealed no acute traumatic injuries, especially no bleedings or cranial fractures. However, a minor extension of the subarachnoid spaces in concert with involution was observed. The thoracic CT revealed extended pleural effusions and infiltrates in the left upper lope. Moreover, the above-mentioned rib fracture was observed.

## DIAGNOSTIC FOCUS AND ASSESSMENT

After respiratory stabilization CT scans of skull, thorax and abdomen was performed. While CT did not show any abnormalities, there were large pleural effusions on both sides with dystelectases. The left ventricle appeared moderately dilated. The patient was treated initially with empiric antibiotic treatment, diuretics, and heart failure medication. Antibiotic treatment was initially escalated to Imipeneme, but terminated when several samples failed to show any bacterial growth. Maximal C-reactive protein before transfer was 162 mg/L and PCT peaked at 0.41 ng/mL. There was no fever. After 2 weeks, sedation was terminated and subsequently the patient woke up but now showed spastic tetraparesis of unknown reason. The patient was transferred to the neurological intensive care unit.

## THERAPEUTIC FOCUS AND OUTCOMES

Due to the dominant neurological symptoms revealing a suspected spastic tetraparesis brainstem stroke and cervical spinal origin were ruled out by radiological diagnostics. Cerebrospinal fluid (CSF) showed no abnormal finding concerning cell count, bacterial culture, protein, and glucose. Due to renewed clinical examination strong rigor and akinesis seemed a possible alternative interpretation of the neurological symptoms. There was no diagnosis of Parkinson's syndrome in the patient's history. We treated with Parkinson's medication (L-Dopa, Dopamine agonists) with an adequate response and definite improvement concerning neurological symptoms. Physiotherapy was performed regularly documenting the progress. So far, no specific treatment is available for the management of HBoV infections, thus no antiviral treatment was initiated.

## DISCUSSION

The HBoV is an ubiquitous pathogens with worldwide distribution that affects all age groups.^12–17^ Although it mainly is associated with respiratory infections in children, severe clinical courses have been described also in adults, but have not yet been systematically investigated.^12–17^ A rare side effect of the HBoV infection could be neurological symptoms that were previously reported in cases of childhood meningitis but also in adults.^18–20^ However, in those earlier studies, no clear association between an active HBoV replication as observed in our patient and the detection of HBoV compartments in the CSF was found, and prognosis of such cases is unclear due to the lack of an appropriate number of reported studies and cases. No typical signs of meningitis were observed in our patient in CSF, clinically and by radiological diagnostics, yet this is not obligatory. Thus, it could also be that the acute injuries resulted from the fact that the patient was weakened by the HBoV associated pneumonia rather than a direct involvement of HBoV in the neurological disorder. Nevertheless, there is a high chance that the active and serious pneumonia that in fact was deduced to the HBoV mono-infection was linked to the neurological symptoms directly or indirectly, and thus the case strongly supports the request for further cohort studies that systematically and controlled address the question if a correlation between the HBoV infection and neurological disorders exists.^20^ Especially the fact that the patient had an underlying neurological disease gives raise to the hypothesis that those patients are more likely affected of injuries as the HBoV infection could weaken the patient and lead to a loss of body control. Most recently, a further atypical case of HBoV infection was observed that was associated with encephalopathy,^21^ thus further confirming that at the present stage neurological side effects of respiratory HBoV infections cannot be excluded, although the causative relation has to be further investigated. Unfortunately, no CSF fluid was left for further analyses, and that is exactly why this case is extremely important. Especially in adult patients, several rare but possible etiologies are not routinely tested such as HBoV and human metapneumovirus, although these pathogens were previously shown to be associated with a distinct clinical entity. Thus, this case is an alarm signal and reminder to test also for HBoV in case of unclear neurological symptoms. In view of the recent literature and taking into account the fact that HBoV was detected in BAL and serum, it is the most likely explanation for the acute neurological exacerbation, although it could also be a simple co-incidence.

Anyway, in the present case it can be assumed that the HBoV pneumonia led to the collateral damage in form of a fresh rib fracture when the emergency situation had risen. This injury could have been avoided if the HBoV infection would have been recognized earlier, symptomatically treated and screened until symptoms would have vanished in this elderly patient.

## INFORMED CONSENT

According to a vote from the Ethical Committee of the University of Witten/Herdecke (vote 75/2013) it was permitted to refrain from the informed consent as the case was reported retrospectively, it was impossible to contact the patient or its relatives, and as the case is presented in double pseudonymized form.

## References

[1] Allander T Tammi MT Eriksson M Cloning of a human parvovirus by molecular screening of respiratory tract samples. *Proc Natl Acad Sci USA* 2005; 102:12891–12896. 16118271 10.1073/pnas.0504666102 PMC1200281

[2] Dina J Vabret A Gouarin S Detection of human bocavirus in hospitalised children. *J Paediatr Child Health* 2009; 45:149–153. 19210599 10.1111/j.1440-1754.2008.01442.x PMC7167115

[3] Longtin J Bastien M Gilca R Human bocavirus infections in hospitalized children and adults. *Emerg Infect Dis* 2008; 14:217–221. 18258113 10.3201/eid1402.070851 PMC2600186

[4] Lusebrink J Schildgen V Tillmann RL Detection of head-to-tail DNA sequences of human bocavirus in clinical samples. *PLoS ONE* 2011; 6:e19457. 21573237 10.1371/journal.pone.0019457 PMC3087758

[5] Schildgen O Human bocavirus: lessons learned to date. *Pathogens* 2013; 2:1–12. 25436878 10.3390/pathogens2010001 PMC4235705

[6] Schildgen O Muller A Allander T Human bocavirus: passenger or pathogen in acute respiratory tract infections? *Clin Microbiol Rev* 2008; 21:291–304.table of contents. 18400798 10.1128/CMR.00030-07 PMC2292574

[7] Schildgen V Khalfaoui S Schildgen O Human bocavirus: from common cold to cancer? Speculations on the importance of an episomal genomic form of human bocavirus. *Rev Med Microbiol* 2014; 25:113–118.

[8] Schildgen V Malecki M Tillmann RL The human bocavirus is associated with some lung and colorectal cancers and persists in solid tumors. *PLoS ONE* 2013; 8:e68020. 23826357 10.1371/journal.pone.0068020 PMC3694905

[9] Windisch W Schildgen V Malecki M Detection of HBoV DNA in idiopathic lung fibrosis, Cologne, Germany. *J Clin Virol* 2013; 58:325–327. 23806665 10.1016/j.jcv.2013.05.020 PMC7172137

[10] Kapoor A Hornig M Asokan A Bocavirus episome in infected human tissue contains non-identical termini. *PLoS ONE* 2011; 6:e21362. 21738642 10.1371/journal.pone.0021362 PMC3125170

[11] Zhao H Zhao L Sun Y Detection of a bocavirus circular genome in fecal specimens from children with acute diarrhea in Beijing, China. *PLoS ONE* 2012; 7:e48980. 23133667 10.1371/journal.pone.0048980 PMC3487788

[12] Edner N Castillo-Rodas P Falk L Life-threatening respiratory tract disease with human bocavirus-1 infection in a 4-year-old child. *J Clin Microbiol* 2012; 50:531–532. 22135260 10.1128/JCM.05706-11 PMC3264148

[13] Jartti L Langen H Soderlund-Venermo M New respiratory viruses and the elderly. *Open Respir Med J* 2011; 5:61–69. 21760867 10.2174/1874306401105010061 PMC3134957

[14] Jartti T Jartti L Ruuskanen O New respiratory viral infections. *Curr Opin Pulm Med* 2012; 18:271–278. 22366993 10.1097/MCP.0b013e328351f8d4

[15] Korner RW Soderlund-Venermo M van Koningsbruggen-Rietschel S Severe human bocavirus infection, Germany. *Emerg Infect Dis* 2011; 17:2303–2305. 22172367 10.3201/eid1712.110574 PMC3311181

[16] Schildgen O Qiu J Soderlund-Venermo M Genomic features of the human bocaviruses. *Future Virol* 2012; 7:31–39. 22389649 10.2217/fvl.11.136 PMC3291126

[17] Soderlund-Venermo M Lahtinen A Jartti T Clinical assessment and improved diagnosis of bocavirus-induced wheezing in children, Finland. *Emerg Infect Dis* 2009; 15:1423–1430. 19788810 10.3201/eid1509.090204 PMC2819894

[18] Mitui MT Tabib SM Matsumoto T Detection of human bocavirus in the cerebrospinal fluid of children with encephalitis. *Clin Infect Dis* 2012; 54:964–967. 22238160 10.1093/cid/cir957

[19] Mori D Ranawaka U Yamada K Human bocavirus in patients with encephalitis, Sri Lanka, 2009–2010. *Emerg Infect Dis* 2013; 19:1859–1862. 24188380 10.3201/eid1911.121548 PMC3837659

[20] Deresinski S Encephalitis and bocavirus? *Clin Infect Dis* 2014; 58: iii. 24571009

[21] Akturk H Sik G Salman N Atypicalpresentation of human bocavirus: severe respiratory tract infection complicated with encephalopathy. *J Med Virol* 2015. 10.1002/jmv.24263 PMC7166798 25966820

